# A Possible Role of Insertion Sequence IS*1216V* in Dissemination of Multidrug-Resistant Elements MES_PM1_ and MES_6272-2_ between *Enterococcus* and ST59 *Staphylococcus aureus*

**DOI:** 10.3390/microorganisms8121905

**Published:** 2020-11-30

**Authors:** Yu-Tzu Lin, Sung-Pin Tseng, Wei-Wen Hung, Chen-Chia Chang, You-Han Chen, Ya-Ting Jao, Yen-Hsu Chen, Lee-Jene Teng, Wei-Chun Hung

**Affiliations:** 1 Department of Medical Laboratory Science and Biotechnology, China Medical University, Taichung 404333, Taiwan; yutzulin@mail.cmu.edu.tw; 2 Department of Medical Laboratory Science and Biotechnology, College of Health Sciences, Kaohsiung Medical University, Kaohsiung 807378, Taiwan; tsengsp@kmu.edu.tw; 3 Division of Endocrinology and Metabolism, Department of Internal Medicine, Kaohsiung Medical University Hospital, Kaohsiung Medical University, Kaohsiung 807377, Taiwan; hung4488@ms57.hinet.net; 4 Department of Microbiology and Immunology, College of Medicine, Kaohsiung Medical University, Kaohsiung 807378, Taiwan; wacsun@gmail.com (C.-C.C.); hady85427@gmail.com (Y.-H.C.); 5 Infection Control Center, Kaohsiung Medical University Hospital, Kaohsiung 807377, Taiwan; ya-tine@yahoo.com.tw; 6 Division of Infectious Diseases, Department of Internal Medicine, Kaohsiung Medical University Hospital, Kaohsiung Medical University, Kaohsiung 807377, Taiwan; infchen@gmail.com; 7 School of Medicine, Graduate Institute of Medicine, Sepsis Research Center, Center of Tropical Medicine and Infectious Diseases, Kaohsiung Medical University, Kaohsiung 807378, Taiwan; 8 Department of Biological Science and Technology, College of Biological Science and Technology, National Chiao Tung University, Hsinchu 300093, Taiwan; 9 Institute of Medical Science and Technology, National Sun Yat-sen University, Kaohsiung 804201, Taiwan; 10 Department of Clinical Laboratory Sciences and Medical Biotechnology, National Taiwan University College of Medicine, Taipei 100229, Taiwan; ljteng@ntu.edu.tw; 11 Department of Medical Research, Kaohsiung Medical University Hospital, Kaohsiung 807377, Taiwan

**Keywords:** *Staphylococcus aureus*, enterococci, IS*1216V*, gene transfer

## Abstract

Sequence type 59 (ST59) is the dominant type of community-associated methicillin-resistant *Staphylococcus aureus* (MRSA) in Taiwan. Previously, we reported that ST59 MRSA harbors enterococcal IS*1216V*-mediated multidrug-resistant composite transposons MES_PM1_ or MES_6272-2_. The MES were found to have a mosaic structure, largely originating in enterococci and partly native to *S. aureus*. The current study aimed to track the origin of the MES and how they disseminated from enterococci to ST59 *S. aureus*. A total of 270 enterococcal isolates were analyzed, showing that two ST64 *Enterococcus faecalis* isolated in 1992 and 11 clonal complex 17 *Enterococcus faecium* harbored MES_PM1_-like and MES_6272-2_-like structures, respectively. Sequence analysis revealed that ST64 *E. faecalis* strain N48 acquired the MES_PM1_-like structure on the plasmid pEflis48. The pEflis48 harbored the enterococci-originated region (erythromycin, kanamycin, and streptomycin resistances) and the *S.* *aureus*-originated region (chloramphenicol resistance) of MES_PM1_ but was separated by the replication region of the plasmid. Homologous recombination between the two direct repeats of IS*1216V* resulted in excision of the replication region of the plasmid to regenerate MES_PM1_. The p4780-1 and pV19 of *E. faecium* carried MES_6272-2_-like structures with IS*1216V*, albeit with multiple insertions by other insertion sequences. The findings show that IS*1216V* plays important roles in bidirectional gene transfer of multidrug resistance between enterococci and *S. aureus*.

## 1. Introduction

Sequence type 59 (ST59) methicillin-resistant *Staphylococcus aureus* (MRSA) is the dominant type of community-associated MRSA (CA-MRSA) in Taiwan and can be divided into two clones: Panton–Valentine leukocidin (PVL)-positive/staphylococcal cassette chromosome *mec* (SCC*mec*) V and PVL-negative/SCC*mec* IV [1,2,3]. Previously, we reported that ST59 *S. aureus* acquires IS*1216V*-mediated enterococcal composite transposons MES, which are responsible for multidrug resistance [4,5]. ST59 PVL-positive/SCC*mec* V MRSA acquires MES_PM1_, which confers resistances to erythromycin, kanamycin, streptomycin, and chloramphenicol; ST59 PVL-negative/SCC*mec* IV MRSA usually acquires MES_6272-2_, which confers resistances to erythromycin, kanamycin, gentamicin, and chloramphenicol. Both the MES_PM1_ and MES_6272-2_ are inserted into the chromosomal *sasK* gene with an 8 bp *att* sequence and are flanked by direct repeats of IS*1216V* at both ends, indicating that MES_PM1_ and MES_6272-2_ are composite transposons mediated by IS*1216V* [4,5]. IS*1216V*, belonging to the IS*6*/IS*26* family, is 809 bp in length with 18 inverted repeats [6,7]. Although IS*1216V* is an enterococcal insertion sequence rarely found in *S. aureus*, up to five copies of IS*1216V* are located in MES_PM1_ and MES_6272-2_ of ST59 *S. aureus* [6].

Regarding multidrug resistance in ST59 *S. aureus*, four resistance determinants originate in enterococci, including *ermB* (erythromycin resistance), *aph(3*′*)-IIIa* (kanamycin resistance), *aadE* (streptomycin resistance), and *aacA-aphD* (gentamicin resistance), while the *cat* gene (chloramphenicol resistance) and its surrounding genetic environment is native to *S. aureus*. MES_PM1_ includes *ermB, aph(3*′*)-IIIa*, *aadE*, and *cat*, while MES_6272-2_ includes *ermB*, *aph(3*′*)-IIIa*, truncated *aadE*, *aacA-aphD*, and *cat*. On the left side of MES_PM1_, the IS*1216V*-*ermB*-[*aph(3*′*)-IIIa*]-*aadE* cluster displays 100% DNA sequence similarity to the corresponding region in pLG2 of *Enterococcus faecalis* [5]. On the right side of MES_PM1_, the *cat* gene and its surrounding environment show high DNA sequence similarity (>99.5%) to the corresponding region of the SAP084A plasmid in *S. aureus*, albeit with four copies of IS*1216V* nearby [5]. The genetic organization of MES_6272-2_ is similar to that of MES_PM1_ except with disruption of the *aadE* gene by *aacA-aphD* and replacement of the region downstream of the *aadE* gene by group II introns [4].

*E. faecalis* and *Enterococcus faecium* are frequently encountered multidrug-resistant microorganisms and have rapidly emerged as troublesome pathogens causing nosocomial infections [8,9,10,11]. Mobile genetic elements (MGEs), including plasmids and transposons, have been shown to play a major role in dissemination of antibiotic resistance among the *Enterococcus* species [12,13]. Some enterococcal MGEs have even been shown to be transferrable to other pathogenic species, such as *Staphylococcus aureus*. Tn*1546*, which encodes vancomycin resistance via *vanA*, originates in enterococci and can transfer to *S. aureus*, leading to emergence of vancomycin-resistant *S. aureus* (VRSA) [12,14]. The structure of Tn*1546* displays heterogeneity, such as an enterococcal insertion sequence IS*1216V* inserted into the backbone [15]. IS*1216V* is also considered to be part of a large mobile element containing Tn*1546* and may cotransfer with Tn*1546* to other species [16].

MES_PM1_ and MES_6272-2_ in ST59 MRSA, the composite transposons mediated by IS*1216V*, are examples of insertion sequences playing a pivotal role in the dissemination of antibiotic resistance. DNA sequence analysis revealed that more than half of the structures originate in enterococci, not in staphylococci. In the current study, we characterized enterococcal clinical isolates with MES-like structures and found evidence of IS*1216V*-mediated interspecies transfer of multidrug resistance genes between staphylococci and enterococci.

## 2. Materials and Methods

### 2.1. Bacterial Isolates

A total of 270 enterococcal isolates collected from clinical specimens were included in this study: (i) 95 randomly selected isolates collected between 1991 and 1993 at the National Taiwan University Hospital (NTUH), (ii) 26 randomly selected isolates from blood cultures collected between 2002 and 2003 at NTUH, and (iii) 149 isolates collected from January 2013 to October 2014 at the Kaohsiung Medical University Hospital (KMUH). Among the 149 isolates, E1–E51 (*n* = 50) were randomly selected vancomycin-susceptible enterococci collected from blood cultures, while V1–V99 (*n* = 99) were first identified as vancomycin-resistant enterococci and collected within the period. Enterococcal multiplex PCR or *groEL* sequencing was used for species identification [17]. Detailed information about the bacterial strains is shown in Table S1.

### 2.2. PCR Mapping of MES Structures

The resistance determinants *ermB*, *aph(3*′*)-IIIa*, *aadE*, *aacA-aphD*, and *cat* were detected as previously described [4]. For *ermB*-, *aph(3*′*)-IIIa*-, and *aadE*-positive isolates, six primer sets (I–VI) were used to screen the MES structures. The entire region of MES_PM1_-like structures in the two *E. faecalis* strains was further mapped using two PCR primer sets (A and B) with the methods we had previously described [5]. Information about the primers is indicated in Table 1, and the locations of the primers are indicated in Figure S1.

### 2.3. Antimicrobial Susceptibility Testing

Susceptibility testing of vancomycin, erythromycin, kanamycin, streptomycin, gentamicin, and chloramphenicol was performed by the agar dilution method according to the 2020 guidelines of the Clinical and Laboratory Standards Institute [18]. *E. faecalis* ATCC 29,212 was used as the reference strain. High-level resistance of streptomycin and gentamicin was defined as minimal inhibitory concentrations of >2000 and >500 mg/L, respectively.

### 2.4. Multilocus Sequence Typing (MLST)

MLST of the 11 MES-carrying enterococcal isolates (2 *E. faecalis* and 11 *E. faecium*) was performed as previously described [19,20]. Sequence types (STs) were assigned according to the program on the MLST website (https://pubmlst.org). Clonal complex (CC) was defined by geoBURST [21].

### 2.5. Pulsed-Field Gel Electrophoresis (PFGE)

PFGE was performed as previously described [22]. In brief, agarose-embedded bacterial DNA was digested with SmaI (New England BioLabs, Ipswich, MA, USA) and then was separated using a CHEF-DRIII apparatus (Bio-Rad Laboratories, Hercules, CA, USA). PFGE was carried out at 200 V and 14 °C for 22.5 h, with the pulse times ranging from 5 to 35 s. The pulsotypes were analyzed by BioNumerics software (Applied Maths, Sint-Martens-Latem, Belgium). The dendrogram of pulsotype relationships was produced by the unweighted pair group method using arithmetic averages (UPGMA) based on Dice similarity indices.

### 2.6. S1 Nuclease-Digested PFGE

S1 nuclease-digested PFGE analysis was performed as previously described to determine the sizes of the plasmids [23]. The agarose-embedded bacterial DNA was incubated at 37 °C for 45 min with 10 units of *Aspergillus oryzae* S1 nuclease (Invitrogen). The reaction was stopped by transferring the agarose-embedded bacterial DNA to ES buffer (0.5 mM EDTA, 1% (*w*/*v*) Sarkosyl, pH 9.0) at 4 °C for 10 min. The plugs were applied to wells of 1.2% (*w*/*v*) agarose gels (Bio-Rad) and run in a CHEF-DRIII apparatus (Bio-Rad) with a pulse angle of 120° and pulse times of 45 s for 14 h and 25 s for 6 h at 200 V in 0.5× Tris-borate-EDTA (TBE). Each band was considered a unit length linear plasmid.

### 2.7. Southern Blot

The linear plasmids in the gel separated by S1-PFGE were depurinated, denatured, neutralized, and transferred to a Hybond-N+ nylon membrane (GE Healthcare, Chicago, IL, USA) by vacuum-blotting. Hybridization was achieved using the digoxigenin-labeled DNA probe specific for the *aadE* gene generated by PCR. The detection of hybridization was performed using an alkaline-phosphatase-conjugated anti-digoxigenin antibody (Roche Diagnostics GmbH, Mannheim, Germany) and the substrate CSPD (Roche Diagnostics GmbH) according to the manufacturer’s instructions.

### 2.8. Sequencing and Analysis of the MES Structures and Their Adjacent Environments

The sequence of the MES_6272-2_-like structure in *E. faecium* 4780-1 was determined using the primer sets for PCR mapping described above, and extension of the IS*12126V* upstream sequence was achieved by inverse PCR. For *E. faecalis* strain N48 and *E. faecium* V19, Illumina MiSeq 300 bp paired-end sequencing was used to determine the MES_PM1_-like and MES_6272-2_-like structures. Total Illumina sequencing output corresponded to approximately 146- and 301-folds of the N48 and V19 genome size, respectively. Contigs were obtained using de novo assembly with SPAdes v3.10.1 [24]. A total of 92 and 261 contigs were yielded for the N48 and V19 genomes, respectively. The gaps between the contigs were filled up by Sanger sequencing. The open reading frame (ORF) was determined using BLAST. The plasmid family was determined using PlasmidFinder 2.0 [25].

### 2.9. Filter Mating

Filter mating was carried out on BHI agar using *E. faecalis* N48 or *E. faecium* V19 as donors, and *E. faecalis* JH2-2 or *S. aureus* RN2677 as recipients. The donor–recipient mix cultures were placed on a filter with incubation at 37 °C for 24 h, followed by resuspension and shaking in BHI broth with incubation at 37 °C for 1 h. The cells were collected and plated on BHI agar containing 250 mg/L erythromycin, 100 mg/L rifampin, 100 mg/L streptomycin (if donor was *E. faecalis* N48), 100 mg/L gentamicin (if donor was *E. faecium* V19), or 25 mg/L fusidic acid (if recipient was *E. faecalis* JH2-2).

### 2.10. Nucleotide Sequence Accession Numbers

The nucleotide sequences (Supplementary Data) of pEflis48, p4780-1, and pV8919 characterized in this study have been deposited in GenBank under accession numbers MT877066–MT877068.

## 3. Results

### 3.1. Molecular Characteristics of MESPM1- or MES6272-2-Carrying Enterococcal Strains

**Figure 1:**
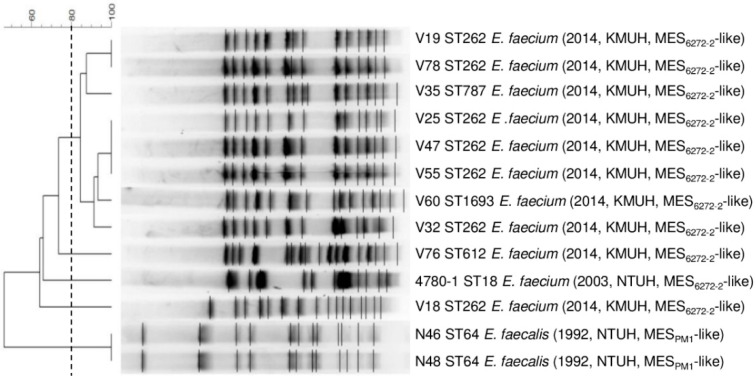
PFGE analysis of the 13 MES-carrying enterococcal strains. The dendrogram was produced by BioNumerics software, showing distance calculated by the Dice similarity index of SmaI-digested DNA fragments. The degree of similarity is shown in the scale. The strain number, ST, species, isolation year, isolation hospital, and MES types are indicated in the figure.

The 270 enterococcal isolates were first screened by PCR for five resistance determinants (*ermB*, *aph(3*′*)-IIIa*, *aadE*, *aacA-aphD*, and *cat*) and antimicrobial susceptibility testing (Table S1). A total of 128 isolates that were positive for *ermB*, *aph(3*′*)-IIIa*, and *aadE* by PCR were selected for further analysis because MES_PM1_ carried the *ermB*, *aph(3*′*)-IIIa*, and *aadE*, while MES_6272-2_ carried *ermB*, *aph(3*′*)-IIIa*, and the nearly complete *aadE* gene with 5′-end disruption by *aacA-aphD*. The PCR mapping of MES structures revealed that two *E. faecalis* strains isolated in 1992 harbored a MES_PM1_-like structure; one *E. faecium* strain isolated in 2003 and 10 *E. faecium* strains isolated from 2013 to 2014 harbored MES_6272-2_-like structures (Table 2). The phylogenetic relatedness of the 13 strains was analyzed by PFGE and MLST (Figure 1). The two *E. faecalis* strains belonged to ST64 and had identical SmaI PFGE patterns. For the 11 *E. faecium* strains, all of them belonged to clonal complex 17 (CC17) with 5 different STs (ST18, ST262, ST612, ST787, and ST1693). The *E. faecium* strains were all resistant to vancomycin except strain 4780-1 (Table S1). The eight strains were clustered together, distant from V18, V76, and 4780-1 (the only strain isolated in 2003).

### 3.2. Localization of the MESPM1- or MES6272-2-Like Elements in Enterococcal Strains

**Figure 2:**
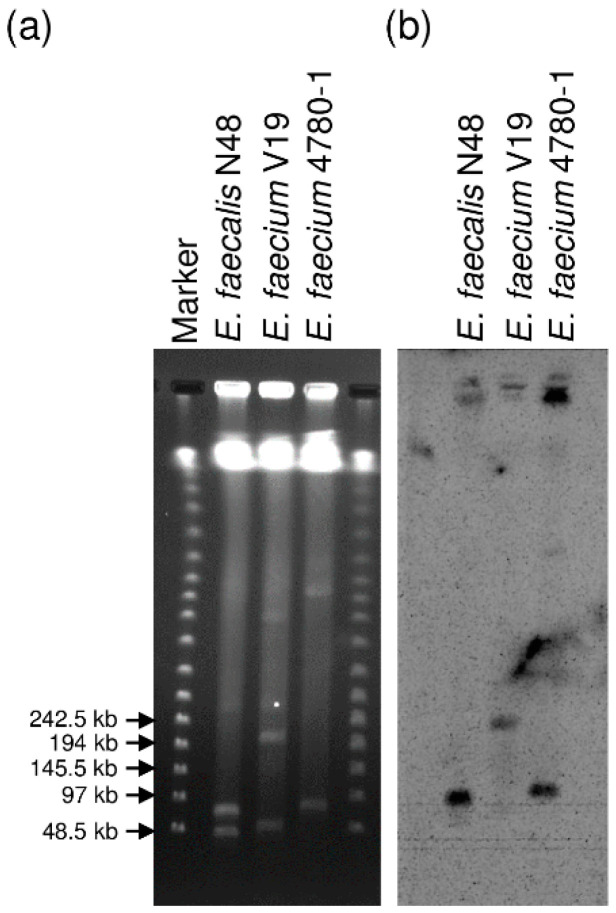
S1 nuclease PFGE analysis followed by Southern blot hybridization with the *aadE* probe of *E. faecalis* N48*, E. faecium* V19, and *E. faecium* 4780-1: (**a**) S1 nuclease-digested DNAs separated by PFGE; (**b**) Southern blot hybridization of the *aadE* probe of the S1 nuclease-digested DNAs.

To determine the location of the MES_PM1_- or MES_6272-2_-like elements in enterococcal strains, *E. faecalis* strain N48 isolated in 1992, *E. faecium* 4780-1 isolated in 2003, and *E. faecium* V19 isolated in 2013 were chosen for S1 nuclease-digested PFGE analysis. The S1 nuclease-digested DNAs were separated by PFGE, followed by Southern blot hybridization with the *aadE* probe (Figure 2). Southern blot hybridization after S1 nuclease-digested PFGE revealed that the MES-like elements of *E. faecalis* strain N48 and *E. faecium* 4780-1 were located on the plasmids with estimated sizes ranging from 48.5 to 97 kb, while the MES_6272-2_-like elements of *E. faecium* V19 were located on a relatively large plasmid with an estimated size ranging from 194 to 242.5 kb. The size of the MES_PM1_-like-carrying plasmid of *E. faecalis* strain 46 and the MES_6272-2_-like-carrying plasmids of *E. faecium* strains V32, V35, V47, and V76 corresponded to *E. faecalis* N48 and *E. faecium* V19, respectively (data not shown).

### 3.3. Sequencing of the MESPM1-Like Elements and the Adjacent Genetic Environments in Enterococcal Strains

**Figure 3:**
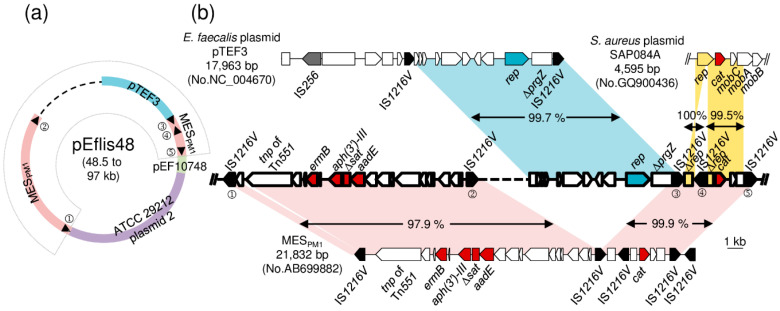
Structure of pEflis48 in *E. faecalis* N48. (**a**) Schematic map of pEflis48 is shown. Blue, pink, green, and purple colors in the circle indicate homologous regions to *E. faecalis* plasmid pTEF3 (GenBank accession number NC_004670), ST59 MRSA MES_PM1_ (GenBank accession number AB699882), *E. faecalis* plasmid pEF10748 (GenBank accession number MK993385), and *E. faecalis* ATCC 29,212 plasmid 2 (GenBank accession number CP008814). The black triangle indicates the position of IS*1216V*. The dashed line in the circle indicates an unresolved sequence. (**b**) Genetic organization of the fan-shaped region indicated in (**a**) is shown. The arrow indicates an open reading frame. The rectangle indicates a truncated gene. Black, grey, red, and blue arrows indicate *tnp* (transposase) of IS*1216V*, *tnp* of IS*256*, resistance determinates, and *rep* (replication initiator) gene, respectively. Homologous regions to *E. faecalis* plasmid pTEF3, *S. aureus* plasmid SAP084A, and MES_PM1_ are shaded in blue, yellow, and red, respectively.

*E. faecalis* N48 was chosen for DNA sequencing to determine the MES-like structure and the surrounding genetic environment. An incomplete plasmid sequence of 50,564 bp in length was determined, and sequence analysis revealed that the MES_PM1_-like structure was located on a mosaic plasmid, pEflis48 (Figure 3a). Positions 1–9944 nt showed 99.7% DNA sequence identity to the corresponding region of the *E. faecalis* plasmid pTEF3. Positions 9136–14,615 and 34,166–50,564 showed 98.4% DNA sequence identity to MES_PM1_ in ST59 MRSA. Positions 14,616–16,723 and 16,724–34,144 showed 99.3% and 99.0% DNA sequence identity to the corresponding regions of the *E. faecalis* plasmid pEF10748 and *E. faecalis* ATCC 29,212 plasmid 2, respectively.

Detailed sequence comparison of the MES_PM1_-like structure in *E. faecalis* N48 is shown in Figure 3b, and the results are highly identical to those for ST59/SCC*mec* V MRSA PM1, except (i) an additional 344 bp sequence near the IS*1216V*①; (ii) an additional region corresponding to *E. faecalis* plasmid pTEF3, including a *rep* gene belonging to the Inc18 family of broad-host-range conjugative plasmid, a truncated gene-encoding pheromone-binding protein, and a copy of IS*1216V*; and (iii) lack of a 298 bp noncoding sequence and an IS*1216V* on the right side of MES_PM1_. Interestingly, the *cat* gene and its surrounding environment that originated in *S. aureus* were also found in pEflis48 and showed 99.9% sequence identity to MES_PM1_. Moreover, PCR and DNA sequencing using the primer set A located in MES_PM1_ (one was located upstream of IS*1216V*② and the other was located downstream of IS*1216V*③, as indicated in Figure 3 and Figure S1) outside the pTEF3 region yielded a 1301 bp amplicon corresponding to a copy of IS*1216V*, indicating that homologous recombination occurred between the two direct repeats of IS*1216V* to excise the pTEF3 region. PCR of *E. faecalis* N46 generated the identical size to that of *E. faecalis* N48 (Figure S1b).

### 3.4. Sequencing of the MES6272-2-Like Elements and the Adjacent Genetic Environments in Enterococcal Strains

**Figure 4:**
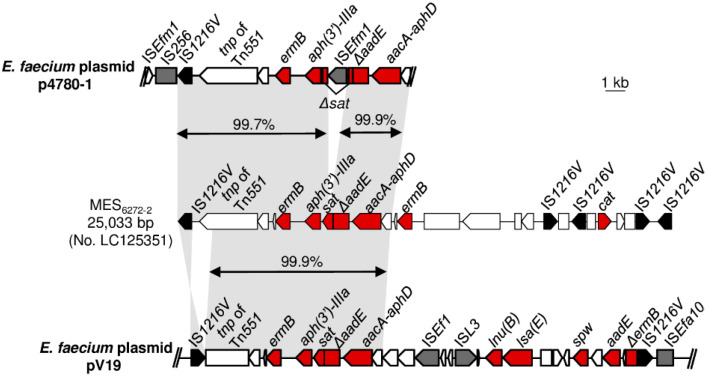
Genetic organization of MES_6272-2_-like structures in p4780-1 and pV19. The arrow indicates an open reading frame. The rectangle indicates a truncated gene. Black, grey, and red colors indicate *tnp* (transposase) of IS*1216V*, *tnp* of other insertion sequences, and resistance determinates, respectively. Homologous regions are shaded.

*E. faecium* 4780-1 and V19 both carried MES_6272-2_-like structures. Sequence analysis revealed that the IS*1216V*-*ermB*-[*aph(3′)-IIIa*]-*sat*-*ΔaadE*-[*aacA-aphD*] cluster of the MES_6272-2_-like structure in *E. faecium* 4780-1 showed >99.7% identity to the corresponding region in ST59/SCC*mec* IV MRSA 6272-2, except an IS*Efm1* insertion into the *sat* gene (Figure 4).

For the MES_6272-2_-like structures of *E. faecium* V19, Illumina sequencing revealed that the MES_6272-2_-like structure was located on plasmid, and 24,402 bp in length was determined. The *ermB*-[*aph(3*′*)-IIIa*]-sat-*ΔaadE*-[*aacA-aphD*] cluster of MES_6272-2_-like structures in *E. faecium* V19 showed 99.9% identity to the corresponding region in ST59/SCC*mec* IV MRSA 6272-2. However, the 3′-end of transposase of Tn*551* was absent in *E. faecium* V19, and the direction of IS*1216V* in front of *ermB* was different between *E. faecium* V19 and ST59/SCC*mec* IV MRSA 6272-2. The region between the *aacA-aphD* gene and the second IS*1216V* of *E. faecium* V19 displayed different structures compared with MES_6272-2_, harboring multiple resistance determinants of *lnu(B)* (lincosamide resistance), *lsa(E)* (pleuromutilin, lincosamide, and streptogramin A resistance), *spw* (spectinomycin resistance), and an *aadE* gene.

### 3.5. Horizontal Transfer of MESPM1 or MES6272-2 Encoding Drug Resistance

Conjugative transfer of MES-like structures between enterococci and *S. aureus* was performed by filter mating. *E. faecalis* N48 or *E. faecium* V19 were used as donors, while *E. faecalis* JH2-2 or *S. aureus* RN2677 were used as recipients. The MES-related resistances were successfully transferred from *E. faecalis* N48 or *E. faecium* V19 to *E. faecalis* JH2-2, with transfer frequencies of 3.7 × 10^−2^ and 2.5 × 10^−7^, respectively. Resistances were transferred to transconjugants (Table 3). All of the transconjugants tested acquired plasmids with sizes similar to their donors detected by S1 nuclease PFGE with Southern blot hybridization. However, no transconjugants were obtained in the cases of transfer from *E. faecalis* N48 or *E. faecium* V19 to *S. aureus* RN2677 (efficiency < 6.5 × 10^−10^).

## 4. Discussion

Previously, we described MES_PM1_ and MES_6272-2_ in ST59 MRSA, the novel multidrug-resistant composite transposons mediated by enterococcal IS*1216V* and mingled with *S. aureus*- and enterococci-originated sequences [4,5]. This raised a question about how the mosaic composite transposons emerged and disseminated into ST59 MRSA, the dominant CA-MRSA in Taiwan identified in 1997 [26]. In the current study, we collected enterococcal clinical isolates from as early as 1991 and analyzed their resistance elements. As expected, MES_PM1_-like and MES_6272-2_-like structures, both surrounded by IS*1216V,* were found in *E. faecalis* and *E. faecium* isolates, respectively. Furthermore, the MES_PM1_-like structure of *E. faecalis* isolated in 1992 had already acquired the *cat* gene and its surrounding genetic environment that was native to *S. aureus*, suggesting that horizontal gene transfer from *S. aureus* to enterococci might have occurred before the emergence of multidrug-resistant ST59 MRSA.

The IS*1216* isoform groups, including IS*1216*, IS*1216E*, and IS*1216V,* are one of the most multiply represented insertion sequences in enterococci and play important roles in the dissemination of resistance determinants [7,12]. They form composite transposons encoding antibiotic resistance in enterococci, such as Tn*5385* (encoding gentamicin, streptomycin, penicillin, erythromycin, and tetracycline/minocycline resistances), Tn5*482*, and Tn*5506* (both encoding vancomycin resistance) [27,28,29]. MES_PM1_ and MES_6272-2_ are rare cases of composite transposons mediated by IS*1216V* found in *S. aureus* [4,5]. We previously reported that MES_PM1_ would be excised, resulting in a single IS*1216V* remaining in MES_PM1_ [4]. The same phenomenon has been demonstrated in pEf37BA of *E. faecium* with the *pbp5* gene surrounded by two direct repeats of IS*1216V* [30]. In the current study, the pEflis48 of ST64 *E. faecalis* carried a MES_PM1_-like structure, within which a region included the *rep* gene and a truncated *prgZ* gene encoding pheromone-binding proteins corresponding to that in pTEF3 of vancomycin-resistant *E. faecalis* (Figure 3). PCR with primer set A located upstream of IS*1216V*② and downstream of IS*1216V*③ generated a 1031 bp amplicon, indicating that RecA-mediated homologous recombination would occur to delete the replication region of pTEF3 and leave a copy of IS*1216V* (Figure 3 and Figure S1). As a result, MES_PM1_, a composite transposon mediated by IS*1216V* highly similar to that in ST59/SCC*mec* V MRSA with erythromycin, kanamycin, streptomycin, and chloramphenicol resistances, would be regenerated. This indicates the important role of IS*1216V* in the dissemination of multidrug resistance between enterococci and ST59 *S. aureus*, the most dominant CA-MRSA in Taiwan.

The *cat* genes found in staphylococci and enterococci are frequently located on small multicopy plasmids and are seldom associated with large multiresistant plasmids [31,32]. In pEflis48 of *E. faecalis* and MES_PM1_ of ST59/SCC*mec*V MRSA, the *cat* gene and its surrounding environments were both highly related to a small *S. aureus* plasmid SAP084A (>99.5% DNA sequence similarity, Figure 3), which indicates that this region originated in *S. aureus*. Furthermore, the *S. aureus*-originated *cat* region in pEflis48 and MES_PM1_ was flanked by two direct repeats of enterococcal IS*1216V* in which another copy embedded, resulting in disruption of the *rep* gene encoding replication initiator of the *S. aureus* plasmid. This implies that the region transferred from *S. aureus* to enterococci, mediated by enterococcal IS*1216V*. Therefore, we hypothesize that the *S. aureus*-originated *cat* region was initially acquired on a large multiresistant plasmid of enterococci by IS*1216V* from a small *S. aureus* plasmid, regenerated to MES_PM1_ by RecA-mediated homologous recombination via two direct repeats of IS*1216V*, and finally disseminated into ST59 *S. aureus*, the dominant CA-MRSA in Taiwan. This would be the first example of IS*1216V*-mediated bidirectional transfer of resistance in *S. aureus* and enterococci.

The MES_PM1_-like structure of *E. faecalis* N48 was located on an Inc18 plasmid. The Inc18 plasmids usually possess the ability to confer antibiotic resistance and to transfer to a variety of Gram-positive cocci including enterococci and staphylococci [12,33]. Inc18 plasmids comprising insertion of Tn*1546* to facilitate dissemination of vancomycin resistance from enterococci to MRSA have been reported [34]. In the current study, although pEflis48 displayed high transfer efficiency (3.7 × 10^−2^/donor) from donor *E. faecalis* N48 to recipient *E. faecalis* JH2-2, it failed to transfer to *S. aureus* RN2677. The reason is unknown and needs to be investigated in the future. A previous study reported that a pSK41-like plasmid is necessary to contribute to successful transfer of the Inc18-like *vanA* plasmid from *E. faecalis* to MRSA [35]. Further research should be carried out to illustrate the transfer mechanism of MES-like structures between enterococci and *S. aureus*.

ST64 *E. faecalis* belongs to CC8. Although CC8 was not included in the major global clusters associated with healthcare-associated infections such as CC2 [8,9], ST64 *E. faecalis* has been reported to be associated with multidrug resistance to erythromycin, kanamycin, streptomycin, gentamicin, and tetracycline [36,37]. In contrast to *E. faecalis*, all of the *E. faecium* isolates characterized in this study were CC17, the most prevalent cluster worldwide, notably in the nosocomial setting [8,38,39]. It has been proposed that progressive evolution to acquire resistance determinants in CC17 *E. faecium* is associated with its success to adapt to conditions of modern hospitals [38]. In the current study, the MES_6272-2_-like structures of the CC17 *E. faecium* strains isolated in 2003, 2013, and 2014 were inserted by several insertion sequences such as IS*Efm1*, IS*Ef1*, and IS*L3*, and an additional cluster of resistance determinants *lnu(B)*, *lsa(E)*, *spw*, and *aadE* was acquired (Figure 4). The unpredictable genomic changes of CC17 *E. faecium* would hamper the tracking of the origin of MES-like structures.

In conclusion, the MES_PM1_ structure with the enterococci-originated region (erythromycin, kanamycin, and streptomycin resistances) and the *S. aureus*-originated region (chloramphenicol resistance) found in ST64 *E. faecalis* and ST59 *S. aureus* indicates that bidirectional gene transfer mediated by IS*1216V* can occur between enterococci and *S. aureus*. Our study is of great importance, as it is the first to demonstrate the role of IS*1216V* in interspecies transfer of multidrug resistance genes between enterococci and ST59 *S. aureus*.

## Figures and Tables

**Table 1:** Primers used in this study.

Primer Set	Primer Name	Sequence (5′ to 3′)	PCR Product Size (bp)
I	IS1216VF	AGTTTACGCACTGCCTCT	2170
	tnpF	CGGTATCCTGGGTGT	
II	IS1216V-fo	CTTCGGTTCATCAAACTGC	1384
	tnp-rev	TCAAATCACCTTCCTACTACCC	
III	tnp-fo	GCGTGTATCTTCGGAGGTA	2727
	ermB-rev	TTGGAACAGGTAAAGGGC	
IV	ermB-fo	ATCTGTGGTATGGCGGGTA	1432
	aphIIIa-rev	ATGACATTGCCTTCTGCG	
V	aphIIIa-fo	TGTCATACCACTTGTCCGC	1345 (2331 if IS*Efm1* insertion)
	aadE-rev	GCTGCCTGGATAGCACATA	
VI	aadE-R	GTTCCCGCCTCTCTTCTA	2254
	aacA-aphD-F	ATACAGAGCCTTGGGAAG	
A	1F	AGTAGCCTTTCCCTCACTT	1301
	35R	GCTTTGACGCTATGACGA	
B	35F	CCTTACCAGTTGTTCCGAA	1619
	LA-R2	CCCATGCAGGTTTCAAAATGTGTAAGTCA	

**Table 2:** Summary of the bacterial strains collected in this study.

	No. of Isolates			
	Isolation Year			Total
	1991–1993	2002–2003	2013–2014	
*Enterococcus* species	95	26	149	270
*E. faecalis*	82	14	30	126
*E. faecium*	10	12	117	139
Others	3 (*E. hirae*)	0	2 (*E. raffinosus*)	5
Positive for *ermB*^+^, *aph3*′*-IIIa*^+^, *aadE*^+^	54	11	63	128
MES structures				
MES_PM1_-like	2 (*E. faecalis*)	0	0	2
MES_6272-2_-like	0	1 (*E. faecium*)	10 (*E. faecium*)	11

**Table 3:** Transfer of drug resistance in filter mating.

	Transfer Frequency	MIC (mg/L)				
		E	KM	SM	GM	C
Recipient: *E. faecalis* JH2-2		0.25	64	128	16	8
Donor: *E. faecalis* N48	3.7 × 10^−2^	>256	>1024	>1024	>1024	64
Transconjugants (*E. faecalis* N48)		>256	>1024	>1024	-	64
Donor: *E. faecium* V19	2.5 × 10^−7^	>256	>1024	32	>1024	8
Transconjugants (*E. faecium* V19)		>256	>1024	-	512	-

Abbreviation: E, erythromycin; KM, kanamycin; SM, streptomycin; GM, gentamicin; C, chloramphenicol. “-”, not detected.

## Supplementary Materials

The following are available online at https://www.mdpi.com/2076-2607/8/12/1905/s1, Figure S1: MES_PM1_, pEflis48, MES_6272-2_, p4780-1, and pV19, Table S1: Information of bacterial strains. Supplementary Data: The nucleotide sequences of pEflis48, p4780-1, and pV8919 characterized in this study.
