# Supplementary material for: A Possible Role of Insertion Sequence IS1216V in Dissemination of Multidrug-Resistant Elements MESPM1 and MES6272-2 between Enterococcus and ST59 Staphylococcus aureus
Source: Microorganisms. 2020 Nov 30;8(12):1905. doi: 10.3390/microorganisms8121905 (PMC7760966; doi:10.3390/microorganisms8121905)
Supplement: Supplementary file 1 [file microorganisms-08-01905-s001.zip › microorganisms-1007591-Supplementary materials/microorganisms-1007591-supplementary table and figure.pdf]

Table S1. Information of bacterial strains

Strain No.	Isolation year	Species	Resistance determiants					PCR mapping of MES						MIC (mg/L)					
			ermB	*aph(3')-IIIa*	*aadE*	*aacA-aphD*	*cat*	I	II	III	IV	V	VI	V	E	KM	SM	GM	C
(i) Isolates collected between 1991 and 1993 in National Taiwan University Hospital																			
14378	1992	*E. faecalis*	+	+	+	+	+	-	-	-	-	+	+	1	>256	>1024	>1024	>1024	32
15508-1	1992	*E. faecalis*	-	-	-	-	-							2	1	64	128	16	8
155010-2	1992	*E. faecalis*	-	-	-	-	-							1	2	64	128	16	8
15518	1992	*E. faecalis*	+	+	+	-	+	+	-	+	+	+	-	2	>256	>1024	>1024	16	32
15965-2	1992	*E. faecalis*	+	+	+	-	-	-	-	-	-	+	-	1	>256	>1024	>1024	16	32
2042	1993	*E. faecalis*	-	-	-	-	-							1	2	64	128	16	16
16124	1992	*E. faecalis*	+	-	-	-	-							1	>256	64	>1024	16	32
27866	1992	*E. faecalis*	+	+	+	-	+	-	-	-	+	$+^{L}$	-	1	>256	>1024	>1024	16	32
24527	1992	*E. faecium*	+	+	+	-	-	-	-	-	-	+	-	1	>256	>1024	>1024	8	16
26530	1992	*E. faecalis*	-	-	-	-	-							2	2	64	128	16	16
23322	1992	*E. faecalis*	+	-	-	-	-							1	>256	64	128	16	32
22746	1991	*E. faecalis*	-	-	-	-	-							4	1	64	128	16	8
21625	1991	*E. faecalis*	+	+	+	-	-	-	-	+	+	+	-	1	>256	>1024	>1024	16	8
21075	1991	*E. faecalis*	-	-	-	-	-							1	1	64	128	16	8
21073	1991	*E. faecalis*	+	+	+	-	+	-	-	-	+	+	-	2	>256	>1024	>1024	16	32
19265	1991	*E. faecalis*	+	+	+	-	-	-	-	-	+	+	-	1	>256	>1024	>1024	16	8
19289	1991	*E. faecalis*	-	-	-	-	+							1	>256	64	>1024	16	16
304	1992	*E. faecalis*	+	+	+	-	-	-	-	+	+	+	-	1	>256	>1024	>1024	16	8
18411	1991	*E. faecalis*	+	+	+	-	-	-	-	-	-	+	-	1	>256	>1024	>1024	16	8
336	1992	*E. faecalis*	-	-	-	-	-							4	1	64	128	16	8
392	1992	*E. faecalis*	-	-	-	-	-							2	2	64	128	16	8
346	1992	*E. faecalis*	-	-	-	-	-							1	2	64	128	16	8
2709	1992	*E. faecalis*	+	-	-	-	-							1	>256	64	128	16	8
2697	1992	*E. faecalis*	+	-	-	-	-							1	>256	64	128	16	8
22245	1992	*E. faecalis*	+	+	+	+	+	-	-	-	-	+	+	1	>256	>1024	>1024	>1024	32
3269	1992	*E. faecalis*	+	+	+	-	-	-	-	+	+	$+^{L}$	-	2	>256	>1024	>1024	16	8
3251	1992	*E. faecalis*	+	-	-	-	-							2	>256	64	128	16	32
2539	1992	*E. faecalis*	-	-	-	-	-							1	2	64	128	16	8
319	1992	*E. faecalis*	+	+	+	-	-	-	-	-	-	+	-	1	>256	>1024	>1024	16	32
21884	1991	*E. faecalis*	-	-	-	-	-							1	2	64	128	16	8
1425	1991	*E. faecalis*	-	-	-	-	-							1	2	64	128	16	8
423	1992	*E. faecalis*	+	-	-	-	+							1	>256	64	128	16	32
1972	1992	*E. faecalis*	-	-	-	-	-							1	1	64	128	16	8
16680	1992	*E. faecalis*	+	+	+	+	+	-	-	-	-	+	+	1	>256	>1024	>1024	>1024	32
18533	1991	*E. faecalis*	+	+	+	+	+	-	-	-	-	+	+	1	>256	>1024	128	>1024	32
20264	1992	*E. faecalis*	+	+	+	+	+	-	-	-	-	+	+	1	>256	>1024	>1024	>1024	32
19950	1992	*E. faecalis*	+	+	+	+	+	-	-	+	+	+	+	2	>256	>1024	>1024	512	64
19955	1992	*E. faecalis*	+	+	+	+	+	-	-	-	-	+	+	1	>256	>1024	>1024	1024	64
1983	1992	*E. faecalis*	+	+	+	+	+	-	-	-	-	+	+	1	>256	>1024	>1024	512	64
1424	1992	*E. faecalis*	-	-	-	-	-							1	0.12	64	256	16	8
14390	1992	*E. faecalis*	+	+	+	+	-	-	-	-	-	+	+	1	>256	>1024	>1024	>1024	8
10088	ND	*E. faecalis*	-	-	-	-	-							1	0.5	64	256	32	8
8039	1992	*E. faecalis*	+	+	+	+	-	-	-	-	-	+	+	1	>256	>1024	>1024	1024	8

11347	1991	*E. faecalis*	+	+	+	+	+	-	-	-	-	+	+	1	>256	>1024	256	>1024	64
2536	1992	*E. faecalis*	+	+	+	+	+	-	-	-	-	+	+	2	>256	>1024	>1024	1024	64
N46	1992	*E. faecalis*	+	+	+	+	+	-	+	+	+	+	-	1	>256	>1024	>1024	>1024	64
2535	1992	*E. faecalis*	+	+	+	+	+	-	-	-	-	+	+	1	>256	>1024	>1024	1024	64
N48	1992	*E. faecalis*	+	+	+	+	+	-	+	+	+	+	-	1	>256	>1024	>1024	>1024	64
16672	1992	*E. faecalis*	+	+	+	+	-	-	-	-	-	+	-	1	16	>1024	>1024	>1024	128
15882-2	1992	*E. faecalis*	+	+	+	+	+	-	-	-	-	+	+	1	>256	>1024	>1024	>1024	64
10138	ND	*E. faecalis*	+	-	-	+	-							1	16	>1024	256	>1024	8
15278	1992	*E. faecalis*	-	-	-	+	-							1	0.25	>1024	256	>1024	8
19126	1991	*E. faecalis*	+	+	+	+	+	-	-	+	+	+	+	1	>256	>1024	256	>1024	128
15498	1992	*E. faecalis*	+	+	+	+	+	-	-	+	+	+	+	1	>256	>1024	>1024	>1024	64
8910	1991	*E. faecalis*	+	+	+	+	+	-	-	-	-	+	+	1	>256	>1024	>1024	1024	64
2264	1992	*E. faecalis*	+	+	+	+	+	-	-	-	-	+	+	1	>256	>1024	>1024	1024	64
25492	1992	*E. faecium*	+	+	+	-	+	-	-	-	+	$+^{L}$	-	0.5	16	>1024	>1024	8	16
25675	1992	*E. faecalis*	-	-	-	-	-							2	1	64	256	32	8
25709	1992	*E. faecalis*	-	-	-	-	-							1	1	64	256	16	8
3280	1992	*E. faecalis*	+	+	+	+	+	-	-	-	+	+	+	2	>256	>1024	>1024	1024	64
22239	1991	*E. faecalis*	+	+	+	+	-	-	-	+	+	+	+	1	>256	>1024	256	>1024	8
14357	1992	*E. faecalis*	+	+	+	+	+	-	-	-	-	+	+	1	>256	>1024	>1024	1024	64
3093	1992	*E. faecalis*	+	+	+	+	+	-	-	-	-	+	+	1	>256	>1024	>1024	>1024	64
15905-1	1992	*E. faecalis*	+	+	+	+	+	-	-	-	-	+	+	1	>256	>1024	>1024	1024	64
15948-2	1992	*E. faecalis*	+	+	+	+	-	-	-	-	-	+	+	1	>256	>1024	>1024	1024	64
19562	1991	*E. faecalis*	+	-	-	-	-							2	>256	>1024	>1024	128	64
22749	1992	*E. faecalis*	+	+	+	+	-	-	-	-	-	+	-	1	>256	>1024	>1024	>1024	64
23379	1992	*E. faecalis*	+	+	+	+	-	-	-	-	-	+	+	1	>256	>1024	>1024	1024	64
24145	1992	*E. faecium*	+	+	+	-	+	-	-	-	-	+	-	0.5	>256	>1024	>1024	8	8
24142	1992	*E. faecalis*	-	-	-	-	+							1	1	64	256	16	8
24959	1992	*E. faecalis*	+	+	+	-	-	-	-	+	+	$+^{L}$	-	2	>256	>1024	>1024	16	8
27238	1992	*E. hirae*	-	-	-	-	+							0.5	0.06	32	128	8	8
26540	1992	*E. faecalis*	-	-	-	-	+							1	1	128	128	16	8
27568	1992	*E. faecalis*	+	+	+	-	-	-	-	+	+	$+^{L}$	-	2	>256	>1024	>1024	32	8
27993	1992	*E. faecalis*	-	-	-	-	-							2	2	128	128	32	8
25711	1992	*E. faecalis*	-	-	-	-	-							2	1	128	>1024	16	4
25850	1992	*E. hirae*	-	-	-	-	-							0.5	0.12	64	64	8	8
24043	1992	*E. faecalis*	+	+	+	-	-	-	-	+	+	+	-	2	32	>1024	>1024	16	64
25966	1992	*E. faecalis*	+	+	+	+	-	-	-	-	-	+	+	2	>256	>1024	>1024	>1024	64
25974	1992	*E. hirae*	-	-	-	-	-							0.5	0.06	64	64	8	8
26457	1992	*E. faecium*	-	-	-	-	+							0.5	2	128	64	8	8
29668	1992	*E. faecalis*	+	+	+	-	-	-	-	-	-	+	-	4	>256	>1024	>1024	32	64
29218	1992	*E. faecalis*	+	+	+	+	-	-	-	-	-	+	+	4	>256	>1024	>1024	>1024	64
29628	1992	*E. faecalis*	-	-	-	-	+							4	2	64	128	32	8
29473	1992	*E. faecium*	+	+	+	-	-	-	-	-	-	+	-	1	>256	>1024	>1024	32	16
28180	1992	*E. faecalis*	+	+	+	-	-	-	-	-	+	+	-	1	>256	>1024	>1024	32	128
28342	1992	*E. faecium*	+	+	+	-	-	-	-	-	-	+	-	1	>256	>1024	>1024	16	32
25911	1992	*E. faecalis*	+	-	-	-	-							2	>256	128	>1024	32	64
29207	1992	*E. faecium*	+	-	-	-	-							2	>256	128	512	8	16
29267	1992	*E. faecalis*	-	-	-	-	-							2	0.5	128	128	32	8

29231	1992	*E. faecalis*	+	-	-	-	-							4	16	128	128	32	8
33780	1993	*E. faecium*	+	+	+	-	-	-	-	-	-	+	-	1	>256	>1024	>1024	16	16
25385	1992	*E. faecalis*	-	-	-	-	-							1	1	128	128	32	8
1980	1993	*E. faecium*	+	+	+	-	+	-	-	-	-	+	-	1	>256	>1024	>1024	16	16
1463	1993	*E. faecium*	+	+	+	-	+	-	-	-	-	+	-	1	>256	>1024	>1024	16	16
(ii) Isolates collected between 2002 and 2003 in National Taiwan University Hospital																			
2766	2003	*E. faecalis*	+	+	+	+	-	-	-	-	-	+	+	1	>256	>1024	64	>1024	16
380-2	2003	*E. faecalis*	+	+	+	+	-	-	-	-	-	+	-	1	>256	>1024	>1024	>1024	64
2658	2003	*E. faecalis*	-	-	-	-	-							4	0.5	64	128	16	16
5646-2	2003	*E. faecalis*	-	+	+	+	-							1	>256	>1024	>1024	>1024	64
7597	2003	*E. faecalis*	-	-	-	-	-							2	2	>1024	128	16	16
6467	2003	*E. faecalis*	-	-	-	-	-							2	2	64	256	16	16
1011	2003	*E. faecalis*	+	+	+	+	-	-	-	-	-	+	+	1	>256	>1024	128	>1024	16
8076	2003	*E. faecalis*	+	+	+	-	-	-	-	-	-	$+^{L}$	-	2	>256	>1024	512	16	64
7263	2003	*E. faecalis*	+	-	-	-	-							1	>256	64	128	16	16
1471	2003	*E. faecalis*	+	+	-	+	-							2	>256	>1024	64	>1024	16
3573	2003	*E. faecalis*	-	-	-	-	-							4	0.5	64	>1024	>1024	16
801-2	2003	*E. faecalis*	+	+	+	+	-	-	-	+	-	+	+	2	>256	>1024	>1024	>1024	64
5393	2003	*E. faecalis*	-	-	-	-	-							8	1	64	512	16	16
3963	2002	*E. faecalis*	-	-	-	-	-							8	0.5	>1024	>1024	512	16
2976	2002	*E. faeclum*	+	+	+	+	-	-	-	-	-	+	+	1	>256	>1024	>1024	>1024	16
6536	2002	*E. faeclum*	+	+	-	+	-							1	>256	>1024	64	>1024	8
3469	2003	*E. faeclum*	+	+	+	+	-	-	-	-	-	+	+	1	>256	>1024	128	>1024	32
539	2002	*E. faeclum*	+	-	-	-	-							1	2	256	64	16	8
8782	2003	*E. faeclum*	+	+	-	+	-							1	>256	>1024	128	>1024	8
9617-2	2002	*E. faeclum*	+	+	+	+	-	-	-	-	-	$+^{L}$	-	2	>256	>1024	>1024	>1024	128
4212	2002	*E. faeclum*	+	+	-	+	-							1	>256	>1024	64	>1024	8
247	2002	*E. faeclum*	+	-	-	-	-							1	2	256	64	16	8
5704	2002	*E. faeclum*	-	-	-	-	-							1	4	256	64	64	8
5791-1	2002	*E. faeclum*	-	+	+	-	-							1	2	>1024	>1024	32	16
4780-1	2003	*E. faeclum*	+	+	+	+	+	-	+	+	+	$+^{L}$	+	2	>256	>1024	>1024	1024	32
9091-2	2002	*E. faeclum*	+	+	+	+	+	-	-	-	+	+	-	>256	>256	>1024	>1024	>1024	32
(iii) Isolates collected in January to October 2014 in Kaohsiung Medical University Hospital																			
E01	2014	*E. faecalis*	-	-	-	-	-							1	0.5	64	128	16	8
E02	2014	*E. faecalis*	-	-	-	+	+							4	1	>1024	>1024	>1024	64
E03	2014	*E. faecalis*	-	-	-	-	-							1	2	64	128	16	8
E04	2014	*E. faecalis*	+	+	+	+	-	-	-	-	-	+	+	1	>256	>1024	64	>1024	8
E05	2014	*E. faecalis*	-	-	-	-	-							1	2	64	128	16	8
E06	2014	*E. faecalis*	-	-	-	-	+							4	0.5	64	128	16	64
E07	2014	*E. faecalis*	-	+	+	+	-							1	>256	>1024	64	>1024	8
E08	2014	*E. faecalis*	+	+	+	+	+	-	-	-	-	+	+	1	>256	>1024	>1024	>1024	32
E09	2014	*E. faecium*	+	+	+	+	-	-	-	-	+	$+^{L}$	-	1	>256	>1024	1024	8	8
E10	2014	*E. faecalis*	+	+	+	+	-	-	-	-	+	+	-	4	>256	>1024	>1024	128	32
E11	2014	*E. faecium*	+	+	+	-	-	-	-	-	+	$+^{L}$	-	1	>256	>1024	>1024	8	8
E12	2014	*E. faecalis*	-	-	-	-	+							4	8	128	128	16	128
E13	2014	*E. faecalis*	+	-	-	-	+							1	>256	64	>1024	8	128
E14	2014	*E. faecium*	+	+	-	+	-							2	>256	>1024	128	>1024	8

E15	2014	*E. faecalis*	+	+	+	-	-	-	-	+	+	+	-	1	>256	>1024	>1024	16	16
E16	2014	*E. faecalis*	+	+	-	+	+							1	>256	>1024	128	16	64
E17	2014	*E. faecalis*	-	-	-	+	-							1	1	>1024	128	>1024	8
E18	2014	*E. faecalis*	+	+	+	+	+	-	-	-	+	+	-	1	>256	>1024	>1024	>1024	16
E19	2014	*E. raffinosus*	-	-	-	-	-							1	0.5	16	64	2	8
E20	2014	*E. faecalis*	+	+	+	+	+	-	-	-	-	+	+	1	>256	>1024	>1024	>1024	64
E21	2014	*E. faecalis*	-	-	-	-	-							1	0.5	64	128	16	8
E22	2014	*E. raffinosus*	-	-	-	-	-							1	0.25	>1024	32	>1024	4
E23	2014	*E. faecalis*	-	-	-	+	-							1	2	>1024	128	>1024	8
E24	2014	*E. faecalis*	+	+	+	+	-	-	-	-	-	+	+	1	>256	>1024	128	>1024	8
E25	2014	*E. faecalis*	+	+	+	+	-	-	-	-	-	+	+	1	>256	>1024	128	>1024	8
E26	2014	*E. faecium*	+	+	+	+	-	-	-	-	+	$+^{L}$	-	1	>256	>1024	1024	>1024	8
E27	2014	*E. faecalis*	+	+	+	+	-	-	-	-	-	+	+	1	>256	>1024	128	1024	8
E28	2014	*E. faecium*	+	-	-	-	-							1	0.5	64	1024	8	8
E29	2014	*E. faecium*	+	-	-	+	-							1	>256	>1024	1024	>1024	8
E30	2014	*E. faecalis*	+	+	+	+	-	-	-	+	+	+	-	1	>256	>1024	>1024	512	8
E31	2014	*E. faecalis*	-	-	-	-	-							1	1	64	128	16	8
E32	2014	*E. faecium*	+	+	+	+	-	-	-	-	+	$+^{L}$	-	1	>256	>1024	512	>1024	8
E33	2014	*E. faecium*	+	+	+	-	-	-	-	-	+	$+^{L}$	-	1	>256	>1024	512	4	8
E34	2014	*E. faecium*	+	+	+	-	-	-	-	-	+	$+^{L}$	-	2	>256	>1024	1024	>1024	8
E35	2014	*E. faecium*	+	+	+	+	-	-	-	-	+	-	-	2	>256	>1024	1024	>1024	8
E36	2014	*E. faecium*	-	-	-	+	-							2	0.25	32	32	4	8
E37	2014	*E. faecium*	+	+	+	+	-	-	-	-	+	$+^{L}$	-	1	>256	>1024	1024	>1024	8
E38	2014	*E. faecalis*	+	+	+	+	+	-	-	-	-	+	+	2	>256	>1024	128	>1024	64
E39	2014	*E. faecalis*	+	+	+	-	-	-	-	-	-	+	-	2	>256	>1024	32	8	8
E40	2014	*E. faecalis*	-	-	-	+	-							2	2	>1024	128	512	8
E41	2014	*E. faecium*	-	-	-	+	-							2	>256	>1024	32	>1024	8
E42	2014	*E. faecium*	+	+	+	+	-	-	-	-	+	$+^{L}$	-	2	>256	>1024	>1024	8	8
E43	2014	*E. faecium*	+	+	+	+	-	-	-	-	+	-	-	2	>256	>1024	1024	>1024	8
E44	2014	*E. faecalis*	+	-	-	-	+							4	>256	128	>1024	8	64
E45	2014	*E. faecalis*	+	+	+	-	-	-	-	+	+	+	-	2	>256	>1024	>1024	256	8
E46	2014	*E. faecalis*	+	+	+	+	-	-	-	-	-	+	+	2	>256	>1024	256	>1024	8
E47	2014	*E. faecium*	-	-	-	-	+							4	2	64	64	4	16
E48	2014	*E. faecium*	-	-	-	-	-							2	2	32	64	4	8
E49	2014	*E. faecalis*	-	-	-	-	-							2	2	128	128	16	8
E51	2014	*E. faecium*	+	-	-	+	-							2	>256	>1024	512	>1024	32
V01	2013	*E. faecium*	+	+	-	+	-							>256	>256	>1024	32	>1024	8
V02	2013	*E. faecium*	+	+	+	+	-	-	-	-	+	$+^{L}$	-	256	>256	>1024	512	>1024	8
V03	2013	*E. faecium*	+	+	-	-	-							>256	256	>1024	32	4	8
V04	2013	*E. faecium*	+	+	-	+	-							256	>256	>1024	32	>1024	8
V05	2013	*E. faecium*	+	+	-	+	-							>256	256	>1024	32	>1024	8
V06	2013	*E. faecium*	+	+	+	-	-	-	-	-	+	-	-	>256	>256	>1024	512	>1024	8
V07	2013	*E. faecium*	+	+	+	+	-	-	-	-	+	-	-	>256	>256	>1024	32	8	8
V08	2013	*E. faecium*	+	+	+	+	-	-	-	-	+	$+^{L}$	-	1	>256	>1024	256	>1024	8
V09	2013	*E. faecium*	+	+	-	-	-							>256	>256	>1024	32	4	8
V10	2013	*E. faecium*	+	+	-	-	-							>256	256	>1024	32	4	8
V11	2013	*E. faecium*	+	+	-	-	-							>256	>256	>1024	32	4	8

V12	2013	*E. faecium*	+	+	+	+	-	-	-	-	+	-	-	256	256	>1024	>1024	>1024	32
V13	2013	*E. faecium*	+	+	-	-	-							>256	>256	>1024	32	4	8
V14	2013	*E. faecium*	+	+	-	-	-							>256	>256	>1024	32	4	8
V15	2013	*E. faecium*	+	+	-	-	-							>256	>256	>1024	32	8	8
V16	2013	*E. faecium*	+	+	-	+	-							>256	>256	>1024	32	>1024	8
V17	2013	*E. faecium*	+	-	-	-	-							>256	256	>1024	32	4	8
V18	2013	*E. faecium*	+	+	+	+	-	+	-	+	+	+	+	>256	>256	>1024	32	>1024	8
V19	2013	*E. faecium*	+	+	+	+	-	+	-	+	+	+	+	>256	>256	>1024	32	>1024	8
V20	2013	*E. faecium*	+	+	-	-	-							256	>256	>1024	32	4	8
V21	2013	*E. faecium*	+	+	-	-	-							>256	>256	>1024	32	4	8
V22	2013	*E. faecium*	+	+	-	+	-							>256	>256	>1024	32	16	8
V23	2013	*E. faecium*	+	+	-	-	-							256	>256	>1024	32	4	8
V24	2013	*E. faecium*	+	+	-	+	-							>256	256	>1024	32	>1024	8
V25	2013	*E. faecium*	+	+	+	+	-	+	-	+	+	+	+	>256	>256	>1024	32	>1024	32
V26	2013	*E. faecium*	+	+	-	-	-							>256	256	>1024	32	8	8
V27	2013	*E. faecium*	+	+	-	+	-							>256	>256	>1024	32	16	8
V28	2013	*E. faecium*	+	+	-	-	-							>256	>256	>1024	32	4	8
V29	2013	*E. faecium*	+	+	-	-	-							>256	>256	>1024	32	4	8
V30	2013	*E. faecium*	+	+	-	-	-							>256	>256	>1024	32	4	8
V31	2013	*E. faecium*	+	+	-	-	-							>256	>256	>1024	32	8	8
V32	2013	*E. faecium*	+	+	+	+	-	+	-	+	+	+	+	>256	>256	>1024	32	>1024	8
V33	2013	*E. faecium*	+	+	-	-	-							>256	>256	>1024	32	4	8
V34	2013	*E. faecium*	+	+	+	+	-	-	-	-	+	$+^{L}$	-	>256	>256	>1024	1024	>1024	8
V35	2013	*E. faecium*	+	+	+	+	-	+	-	+	+	+	$+^{L}$	>256	>256	>1024	32	>1024	8
V36	2013	*E. faecium*	+	+	-	-	-							>256	>256	>1024	32	4	8
V37	2013	*E. faecium*	+	+	-	-	-							>256	>256	>1024	32	4	8
V38	2013	*E. faecium*	+	+	-	+	-							>256	>256	>1024	32	>1024	8
V39	2013	*E. faecium*	+	+	-	-	-							>256	>256	>1024	32	4	8
V40	2013	*E. faecium*	+	+	+	+	-	-	-	-	+	$+^{L}$	-	>256	>256	>1024	1024	>1024	8
V41	2013	*E. faecium*	+	+	-	-	-							>256	>256	>1024	64	8	8
V42	2013	*E. faecium*	+	+	-	-	-							>256	>256	>1024	32	4	8
V43	2013	*E. faecium*	+	+	+	+	-	-	-	-	+	$+^{L}$	-	256	>256	>1024	1024	>1024	8
V44	2013	*E. faecium*	+	+	-	-	-							>256	>256	>1024	32	4	8
V45	2013	*E. faecium*	+	+	-	-	-							>256	>256	>1024	32	4	8
V46	2013	*E. faecium*	+	+	-	-	-							>256	>256	>1024	32	4	8
V47	2013	*E. faecium*	+	+	+	+	-	+	-	+	+	+	+	>256	>256	>1024	32	>1024	8
V48	2013	*E. faecium*	+	+	-	-	-							256	>256	>1024	32	4	8
V49	2013	*E. faecium*	+	+	+	+	-	-	-	-	-	+	-	>256	>256	>1024	>1024	>1024	16
V50	2013	*E. faecium*	+	+	-	+	-							256	>256	>1024	32	>1024	8
V51	2013	*E. faecium*	+	+	+	+	-	-	-	-	-	+	-	>256	>256	>1024	>1024	>1024	16
V52	2013	*E. faecium*	+	+	-	+	-							>256	>256	>1024	32	8	8
V53	2013	*E. faecium*	+	+	-	+	-							>256	>256	>1024	32	>1024	8
V54	2014	*E. faecium*	+	+	-	+	-							256	>256	>1024	32	>1024	8
V55	2014	*E. faecium*	+	+	+	+	-	+	-	+	+	+	+	>256	>256	>1024	32	>1024	8
V56	2014	*E. faecium*	+	+	+	+	-	-	-	-	+	+	-	>256	>256	>1024	32	>1024	8
V57	2014	*E. faecium*	+	+	-	-	-							>256	>256	>1024	32	4	8
V58	2014	*E. faecium*	+	+	+	+	-	-	-	-	-	+	-	>256	>256	>1024	>1024	>1024	16

V59	2014	*E. faecium*	+	+	-	-	-							>256	>256	>1024	64	>1024	8
V60	2014	*E. faecium*	+	+	+	+	-	+	-	+	+	+	+	>256	>256	>1024	64	>1024	8
V61	2014	*E. faecium*	+	+	+	+	-	-	-	-	+	+	-	2	>256	>1024	1024	>1024	8
V62	2014	*E. faecium*	+	+	-	-	-							>256	>256	>1024	32	4	8
V63	2014	*E. faecium*	+	+	-	+	-							256	>256	>1024	32	>1024	8
V64	2014	*E. faecium*	+	+	-	-	-							>256	>256	>1024	32	4	8
V65	2014	*E. faecium*	+	+	-	-	-							>256	>256	>1024	32	2	8
V66	2014	*E. faecium*	+	+	-	-	-							>256	>256	>1024	32	4	8
V67	2014	*E. faecium*	+	+	+	+	-	-	-	-	+	$+^{L}$	-	>256	>256	>1024	512	>1024	4
V68	2014	*E. faecium*	+	+	+	+	-	-	-	-	+	$+^{L}$	-	>256	>256	>1024	1024	64	8
V69	2014	*E. faecium*	+	+	-	-	-							>256	>256	>1024	64	16	8
V70	2014	*E. faecium*	+	+	-	-	+							256	>256	>1024	16	4	32
V71	2014	*E. faecium*	+	+	+	+	-	-	-	-	+	$+^{L}$	-	256	>256	>1024	1024	>1024	8
V72	2014	*E. faecium*	+	+	-	-	-							256	>256	>1024	16	4	8
V73	2014	*E. faecium*	+	+	+	+	-	-	-	-	+	$+^{L}$	-	256	>256	>1024	>1024	8	8
V74	2014	*E. faecium*	+	+	-	+	-							256	>256	>1024	64	8	8
V75	2014	*E. faecium*	+	+	+	+	-	-	-	-	+	$+^{L}$	-	256	>256	>1024	>1024	8	8
V76	2014	*E. faecium*	+	+	+	+	-	+	-	+	+	+	+	>256	>256	>1024	64	>1024	8
V77	2014	*E. faecium*	+	+	+	+	-	-	-	-	+	$+^{L}$	-	256	>256	>1024	256	4	8
V78	2014	*E. faecium*	+	+	+	+	-	+	-	+	+	+	+	>256	>256	>1024	64	>1024	8
V79	2014	*E. faecium*	+	+	-	-	-							256	>256	>1024	32	4	8
V80	2014	*E. faecium*	+	+	-	+	-							256	>256	>1024	64	>1024	8
V81	2014	*E. faecium*	+	+	-	-	-							256	>256	>1024	64	16	8
V82	2014	*E. faecium*	+	+	-	-	-							256	>256	>1024	32	4	8
V83	2014	*E. faecium*	+	+	+	+	-	-	-	-	+	$+^{L}$	-	256	>256	>1024	>1024	8	8
V84	2014	*E. faecium*	+	+	+	+	-	-	-	-	-	+	-	256	>256	>1024	>1024	>1024	32
V85	2014	*E. faecium*	+	+	+	+	-	-	-	-	+	$+^{L}$	-	256	>256	>1024	>1024	>1024	8
V86	2014	*E. faecium*	+	+	+	+	-	-	-	-	+	$+^{L}$	-	>256	>256	>1024	>1024	8	8
V87	2014	*E. faecium*	+	+	+	+	-	-	-	-	+	$+^{L}$	-	256	>256	>1024	1024	256	8
V88	2014	*E. faecium*	+	+	+	+	-	-	-	-	-	+	-	256	>256	>1024	>1024	>1024	32
V89	2014	*E. faecium*	+	+	-	-	-							256	>256	>1024	32	4	8
V90	2014	*E. faecium*	-	+	-	+	-							256	0.25	>1024	32	>1024	8
V91	2014	*E. faecium*	+	+	+	+	-	-	-	-	+	$+^{L}$	-	256	>256	>1024	>1024	>1024	8
V92	2014	*E. faecium*	+	+	-	-	-							256	>256	>1024	32	4	8
V93	2014	*E. faecium*	+	+	-	+	-							>256	>256	>1024	64	>1024	8
V94	2014	*E. faecium*	+	+	+	+	-	-	-	-	+	$+^{L}$	-	>256	>256	>1024	1024	>1024	8
V95	2014	*E. faecium*	+	+	-	+	-							256	>256	>1024	32	>1024	8
V96	2014	*E. faecium*	+	+	-	+	-							256	>256	>1024	32	8	8
V97	2014	*E. faecium*	+	+	-	+	-							256	>256	>1024	64	512	8
V98	2014	*E. faecium*	+	+	+	+	-	-	-	-	+	$+^{L}$	-	256	>256	>1024	1024	>1024	8
V99	2014	*E. faecium*	+	+	+	+	-	-	-	-	+	$+^{L}$	-	>256	>256	>1024	>1024	128	8

ND, no record to isolation year; $+^{L}$: PCR product is longer than predicted size. PCR mapping of MES carried out only for strains with *ermB*, *aph(3')-IIIa* and *aadE*.
Grey shaded, high-level streptomycin or gentamicin resistance detected by brain-heart infusion agar with 500 mg/L gentamicin or 2000 mg/L streptomycin according to the 2019 guidelines of Clinical and Laboratory Standards Institute.
Abbreviations: V, vancomycin; E, erythromycin; KM, kanamycin; SM, streptomycin; GM, gentamicin; C, chloramphenicol.

Figure S1

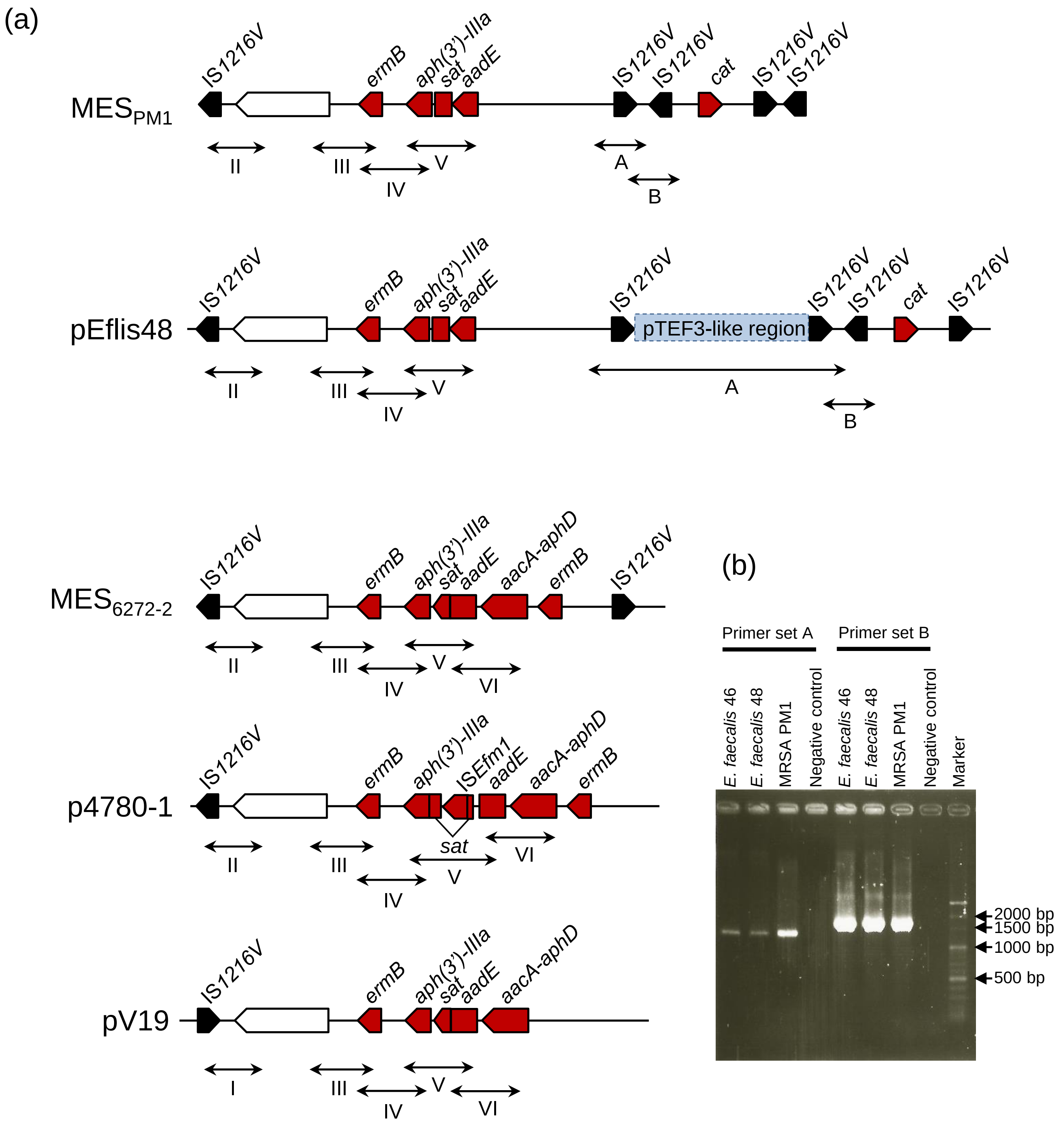

Figure S1. MES_{PM1}, pEflis48, $MES_{6272\text{-}2}$, p4780-1 and pV19. (a) Cartoon representation for PCR mapping. The double arrows indicate the location of PCR primer sets I to VI and a to b as described in Table 2. Resistance determinants are shown in red, and IS*1216V* are shown in black. (b) PCR mapping using primer sets a and b of *E. faecalis* 46 and 48 following 1.5% agarose gel electrophoresis. ST59 MRSA PM1 is positive control. Identical size of the PCR amplicons from *E. faecalis* 46, 48 and MRSA PM1 using primer set a indicated homologous recombination would occur between the two direct repeats of IS*1216V* to delete pTEF3-like region in pEflis48.
